# Bilateral purtscher-like retinopathy associated with DRESS syndrome: a case report

**DOI:** 10.1186/s12348-024-00433-x

**Published:** 2024-09-30

**Authors:** Langxuan Yuan, Yuchen Li, Hanze Zhang, Yucen Hou, Qianyan Kang, Jianqin Lei

**Affiliations:** Department of Ophthalmology, First Affiliated Hospital of Xi’an Jiaotong University, 277# west Yan Ta road, Xi’an, Shaanxi 710061 China

**Keywords:** Purtscher-like retinopathy, DRESS syndrome, Inflammatory storm, Delayed hypersensitivity reaction

## Abstract

**Background:**

To report a case of Bilateral Purtscher-like retinopathy associated with DRESS syndrome managed with ocular and systemic treatments.

**Case presentation:**

A 29-year-old healthy female developed multi-organ (cutaneous, hematologic, renal and hepatic) disfunction and profound vision loss 1 month after Human papillomavirus vaccine injection. At the first presentation, her visual acuity was counting fingers in both eyes. Fundus exam showed remarkable cotton-wool spots, retinal hemorrhages and macular edema. She was diagnosed DRESS syndrome and Purtscher-like retinopathy and treated with intravitreal injection of ranibizumab, systemic steroids anticoagulants, and plasma exchange. The patient finally recovered from this life-threatening condition but left with permanent visual damage.

**Conclusion:**

Purtscher-like retinopathy could be complicated by DRESS syndrome which is usually considered a type IV hypersensitivity reaction.

## Introduction

Purtscher-like retinopathy is a rare eye disease caused by severe but non-traumatic conditions, such as acute pancreatitis, fat embolism syndrome and autoimmune, connective tissue disorders [1]. Drug Reaction with Eosinophilia and Systemic Symptoms (DRESS) syndrome is a life-threatening disease triggered by delayed immunological reactions to drugs, usually accompanied with a transient state of immune suppression and reactivation of latent herpes virus infections. Major clinical manifestations of DRESS include fever, widespread rash facial edema, organ involvement, and hematological abnormalities, including eosinophilia and atypical lymphocytosis [2, 3]. We herein reported a rare case of Purtscher-like retinopathy associated with Dress syndrome suggesting that type IV immunoreactions may contribute to Purtscher-like retinopathy.

## Case report

**Fig. 1:**
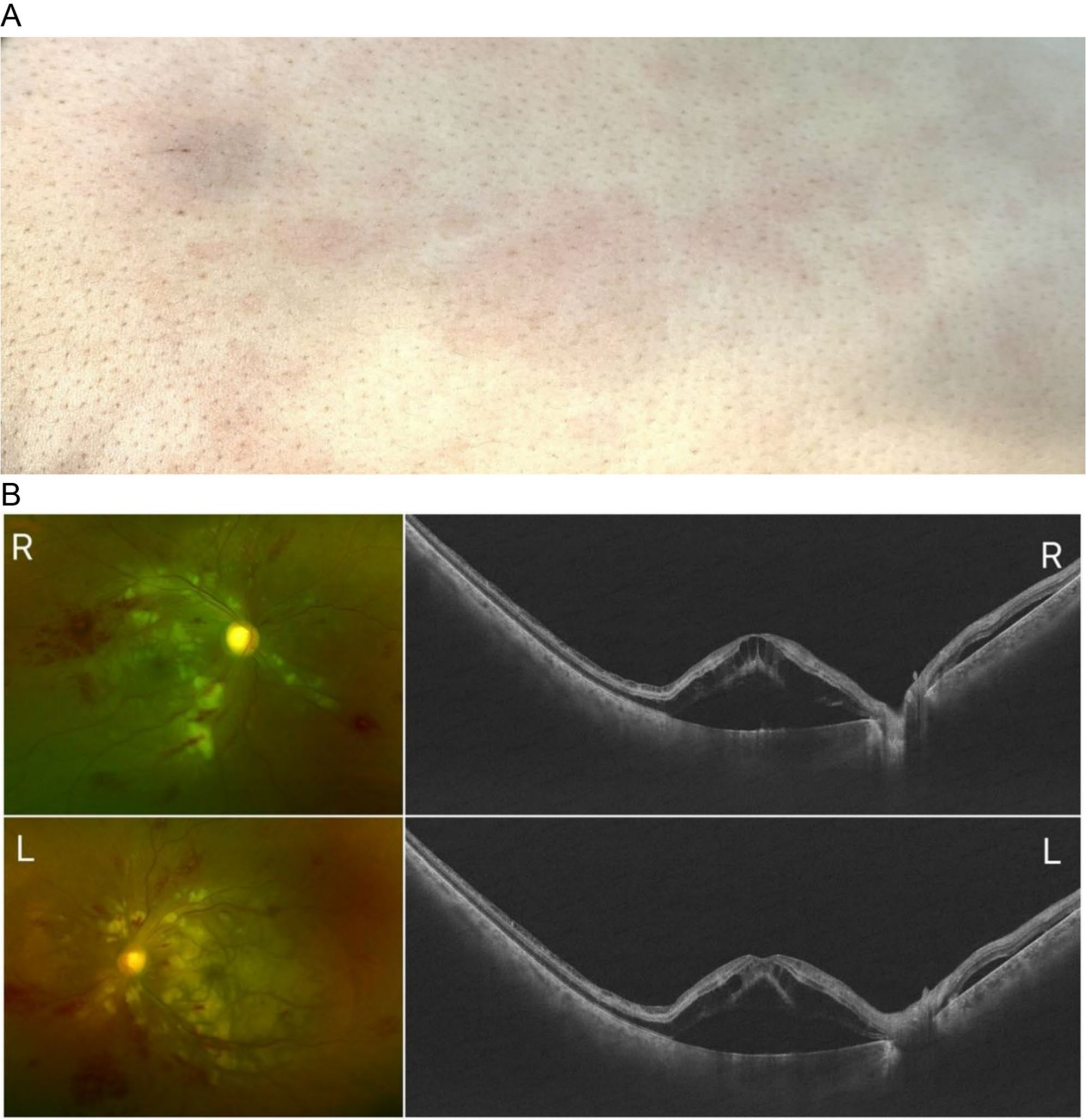
First Presentation of Bilateral Purtscher-Like Retinopathy in a 29-Year-Old Female. **A**: A photo of the patient’s skin. **B**: Bilateral fundus photographs and OCT

A 29-year-old female was referred from the intensive unit care (ICU) due to sudden bilateral vision loss over four days. She received the Human Papillomavirus vaccine one month prior, subsequently developing fever and symmetrical annular erythema(Fig. 1A) on her trunk and limbs ten days later. Laboratory tests revealed Increased systemic inflammatory markers and liver and kidney damage: white blood cell count of 14,030/µL, with 31% cytotoxic T cells and 23.4% B cells. D-dimer levels were at 9.55 mg/L, IL-6 at 30 pg/mL, aspartate aminotransferase at 82 U/L, and creatinine at 198 µmol/L. She had no previous health issues and was diagnosed with DRESS syndrome twenty days post-vaccine.

At the first presentation, her visual acuity was finger count at 10 cm OU, with normal intraocular pressure. The anterior segment was normal, and the vitreous was clear. Dilated fundus examination revealed numerous cotton-wool spots and retinal hemorrhages in the macular and peripapillary regions bilaterally. Optical coherence tomography (OCT) showed severe macular edema and “middle limiting membrane sign” in both eyes. Aqueous humor tapped for next-generation sequencing yielded negative results for pathogens (Fig. 1B). A diagnosis of Bilateral Purtscher-like retinopathy was made, and intravitreal injection of Ranibizumab (IVR) were administered. Systemic treatment in the ICU included the administration of steroids, anticoagulants, plasma exchange, and supportive therapies.

A, Photos of the patient’s skin shows the typical skin manifestations of DRESS syndrome. B, Bilateral fundus photographs showing intraretinal hemorrhages and numerous cotton wool spots.Bilateral OCT B-scans (24 × 20 mm, BM-400 K TowardPi) showing severe macular edema and “middle limiting membrane” sign.

**Fig. 2:**
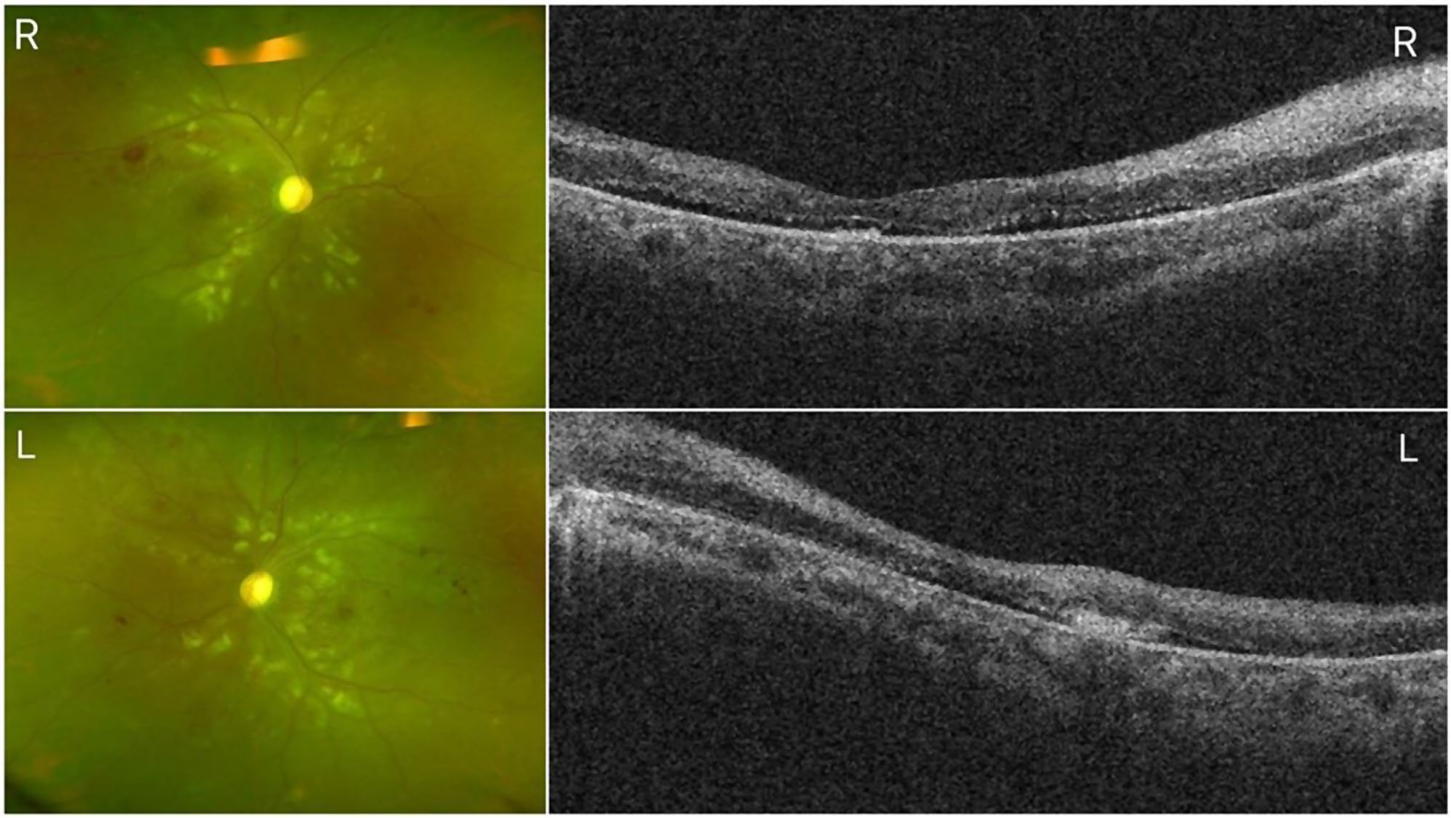
One-Month Follow-up of Bilateral Purtscher-Like Retinopathy in a 29-Year-Old Female

One month post-IVR, the patient was discharged from the ICU with controlled systemic conditions. Her best-corrected visual acuity; improved to 0.12 OD and 0.01 OS. Fundus examination showed significant improvement but residual thinning of the inner retina (Fig. 2).

Bilateral fundus photographs showing much improvement of the retinal lesions.OCT B-Scans (512 × 128 volumes scans, Cirrus HD5000, Zeiss)) showing subtle subretinal fluid and thinning of the inner retina bilaterally.

## Discussion

Purtscher retinopathy is an occlusive microvasculopathy related to trauma first described by Otmar Purtscher. When the condition occurs in the absence of trauma it is referred to as purtscher-like retinopathy [4]. Purtscher-like retinopathy features polygonal retinal whitening and hemorrhages around the optic disc, seen in conditions like acute pancreatitis, fat embolism syndrome, renal failure, childbirth, and connective tissue disorders [5]. Its diagnosis is based on clinical history and presentation. The characteristic findings in the fundus are Purtscher flecken, which are multiple areas of polygonal retinal whitening between the retinal arterioles and venules, usually accompanied by superficial cotton-wool spots at the posterior pole [6]. Those microangiopathic changes may result from occlusions of precapillary arterioles likely induced by complement-mediated leukoembolization and the hypercoagulable state or direct viral infection of the vascular endothelium leading to vasculitis [7]. DRESS syndrome is a severe drug-induced reaction characterized by skin manifestations, internal organ involvement, and hematologic abnormalities [8]. It is often linked to T cell-mediated delayed hypersensitivity and possible human herpesvirus reactivation [9]. In this case, vaccine hypersensitivity and CMV reactivation might trigger a severe immune response, leading to multi-organ and hematologic dysfunction. The resulting inflammatory storm and hypercoagulable state likely caused multiple retinal arteriole occlusions. There are no consensus guidelines for managing this condition, particularly in life-threatening scenarios complicating ocular management [10]. To our knowledge, purtscher-like retinopathy related to DRESS syndrome has rarely been reported before. Our case suggested that Purtscher-like retinopathy could occur in the context of secondary inflammatory storm and type IV immunoreactions induced by drug or vaccine hypersensitivity.

## Data Availability

No datasets were generated or analysed during the current study.

## Abbreviations

DRESS: Drug Reaction with Eosinophilia and Systemic Symptoms
ICU: Intensive Unit Care
OCT: Optical Coherence Tomography
IVR: Intravitreal Injection of Ranibizumab
